# Increased Tumor Immune Microenvironment CD3+ and CD20+ Lymphocytes Predict a Better Prognosis in Oral Tongue Squamous Cell Carcinoma

**DOI:** 10.3389/fcell.2020.622161

**Published:** 2021-02-18

**Authors:** Raísa Sales de Sá, Marisol Miranda Galvis, Bruno Augusto Linhares Almeida Mariz, Amanda Almeida Leite, Luciana Schultz, Oslei Paes Almeida, Alan Roger Santos-Silva, Clovis Antonio Lopes Pinto, Pablo Agustin Vargas, Kenneth John Gollob, Luiz Paulo Kowalski

**Affiliations:** ^1^Department of Oral Diagnosis, Piracicaba Dental School, University of Campinas, Piracicaba, Brazil; ^2^Department of Oral Biology and Diagnostic Sciences, Dental College of Georgia, Augusta University, Augusta, GA, United States; ^3^Department of Anatomic Pathology, Instituto de Anatomia Patologica–IAP, Santa Barbara d'Oeste, Brazil; ^4^Department of Anatomic Pathology, A. C. Camargo Cancer Center, São Paulo, Brazil; ^5^International Research Center, A. C. Camargo Cancer Center, São Paulo, Brazil; ^6^National Institute for Science and Technology in Oncogenomics and Therapeutic Innovation, A.C. Camargo Cancer Center, São Paulo, Brazil; ^7^Department of Head and Neck Surgery and Otorhinolaryngology, A. C. Camargo Cancer Center, São Paulo, Brazil; ^8^Head and Neck Surgery Department, Medical School, University of São Paulo, São Paulo, Brazil

**Keywords:** tumor-infiltrating lymphocytes, immune microenvironment, tongue squamous cell carcinoma (TSCC), CD20 B cells+, CD3

## Abstract

**Background:** Oral tongue squamous cell carcinoma (OTSCC) causes over 350,000 cases annually and particularly impacts populations in developing countries. Smoking and alcohol consumption are major risk factors. Determining the role of the tumor immune microenvironment (TIME) in OTSCC outcomes can elucidate immune mechanisms behind disease progression, and can potentially identify prognostic biomarkers.

**Methods:** We performed a retrospective study of 48 OTSCC surgical specimens from patients with tobacco and alcohol exposures. A panel of immunoregulatory cell subpopulations including T (CD3, CD4, CD8) and B (CD20) lymphocytes, dendritic cells (CD1a, CD83), macrophages (CD68), and immune checkpoint molecules programmed cell death protein 1 (PD-1) and ligand 1 (PD-L1) were analyzed using immunohistochemistry. The levels of immune effector cell subpopulations and markers were analyzed in relation to overall survival.

**Results:** Pathological characteristics of the tumor microenvironment included inflammatory infiltrates (83.3%), desmoplasia (41.6%), and perineural invasion (50.0%). The TIME contained high levels of T cells (CD3+, CD4+, and CD8+) and B cells (CD20+), as well as immature (CD1a) and mature (CD83) dendritic cells, PD-1, and PD-L1. Higher numbers of TIME infiltrating CD3+ T cells and CD20+ B cells were predictive of better survival, while higher levels of CD83+ mature dendritic cells predicted better survival. CD3+ T cells were identified as an independent prognostic marker for OTSCC. Lastly, CD3+ T cells were strongly correlated with the number of CD8+ cells and PD-L1 expression.

**Conclusion:** Our findings provide evidence that the TIME profile of OTSSC impacted prognosis. The high expression of CD3+ T cells and B cells are predictive of better overall survival and indicative of an immunologically active, inflammatory TIME in patients with better survival. The number of CD3+ T cells was an independent prognostic marker.

## Introduction

Oral cancer is among the 10 most frequent cancers worldwide, with an incidence of over 350,000 new cases annually (Bray et al., 2018). Oral squamous cell carcinoma (OSCC) of mucosal origin accounts for more than 90% of cases (Curado et al., 2016; Bray et al., 2018). Patients affected by oral tongue squamous cell carcinoma (OTSCC) are often elderly males over the sixth decade of life, with prolonged exposures to tobacco and alcohol (Scully and Bagan, 2009; Ng et al., 2017). Over half of cases are diagnosed and treated at locally advanced stages, consequently presenting a high risk of recurrences and locoregional metastasis (Ng et al., 2017).

Extensive research has elucidated underlying mechanisms of tumor cell survival, proliferation, and dissemination (Hanahan and Weinberg, 2000). A dynamic signaling network between tumor cells and the tumor microenvironment (TME) significantly influences cancer progression and response to conventional therapies (Hanahan and Weinberg, 2011; Salo et al., 2014). The TME contains varied components including immune cells (e.g., tumor-infiltrating lymphocytes [TILs], macrophages, and dendritic cells); cancer-associated fibroblasts, and endothelial cells; and in addition, an extracellular matrix (fibronectin and collagen fibers) and soluble factors (e.g., enzymes, growth factors, and chemokines) (Kim, 2007; Lim et al., 2018).

The tumor immune microenvironment (TIME) plays a critical role in the recognition and clearance of tumor cells, as well as the generation of detrimental immunosuppressive microenvironments (Munn and Bronte, 2016; Hadler-Olsen and Wirsing, 2019). Immune surveillance is an important process that counters carcinogenesis and maintains homeostasis (Kim, 2007; Ferris, 2015). Growing evidence suggests that the composition of immune cell infiltrates may be a potential prognostic marker in OSCC (Almangush et al., 2020; Perri et al., 2020; Zhou et al., 2020). The immune checkpoint molecule programmed cell death receptor 1 (PD-1) and its ligand, programmed cell death ligand - 1 (PD-L1), can induce immune suppression, thereby promoting the progression of disease, and protecting tumors from immune aggression (Ferris, 2015; Lim et al., 2018). Recognizing the importance of immunological components of tumors, an immunescore was developed that can provide a prognostic factor for global survival, as well as for a means for cancer classification (Galon et al., 2012; Fridman et al., 2013). In addition, tumor immune components provide a target for new therapeutic approaches, including immunotherapy via blocking anti-PD-1/PD-L1 (Moskovitz and Ferris, 2018). Importantly, environmental factors such as tobacco and alcohol consumption can modify the tumor immune profile, especially by suppressing T cell chemotaxis (de la Iglesia et al., 2020). Therefore, we aimed to characterize the TIME from a homogeneous cohort of OTSCC patients with tobacco and alcohol exposures, and to further evaluate its impact on survival outcomes.

## Patients and Methods

### Study Design and Ethical Approval

Surgical specimens of OTSCC from 48 patients treated from 2010 to 2017 were retrieved from the archives of the A.C. Camargo Cancer Center (São Paulo, Brazil) and the Institute of Pathological Anatomy (Santa Bárbara d'Oeste, São Paulo State, Brazil). Eligibility criteria included previously untreated OTSCC and tobacco and alcohol consumption. Patients with previous or other concomitant primary carcinomas were not eligible. Patients' medical records were reviewed to retrieve sociodemographic characteristics (e.g., gender, age, risk habits), clinical data (e.g., clinical stage, tumor local, lymph node metastasis, and distant merastasis), therapeutic modality (surgery, radiotherapy, and/or chemotherapy), and follow-up status (Edge and Compton, 2010). Tumor stage and clinical stage were categorized as initial (I and II) or advanced (III and IV) (Table 1).

**Table 1:** Clinicopathological features of oral squamous cell carcinoma patients.

**Parameters**		
**Age**		
**Range**	**43–86**	
**Mean**	**61.2**	
**Median**	**61.5**	
	***n***	**%**
**Gender**		
M	35	73.0
F	13	27.0
**Tumor size**		
T1	15	31.2
T2	13	27.1
T3	09	18.8
T4	11	22.9
**Lymph node metastasis**		
N0	30	62.5
N1	10	20.8
N2	8	16.7
**Distant metastasis**		
M	0	0
**Clinical stage**		
I	13	27.1
II	10	20.8
III	08	16.7
IV	17	35.4
**Histological grade**		
Well differentiated	16	33.3
Moderately differentiated	25	52.1
Poorly differentiated	7	14.6
**Status**		
Alive	31	64.6
Dead	10	20.8
Lost to follow-up	7	14.6
**Recurrence**		
Yes	17	35.4
No	31	64.6
**Desmoplasia**		
Presente	20	41.6
Absent	2	4.2
NA	26	54.2
**Desmoplasia intensity**		
Weak	9	45
Moderate	7	35
Strong	4	20
**Inflammatory infiltrate**		
Present	40	83.3
Absent	0	0
NA	8	16.7
**Vascular invasion**		
Present	4	8.3
Absent	43	89.6
NA	1	2.1
**Perineural invasion**		
Present	24	50.0
Absent	23	47.9
NA	1	2.1
**Lymphatic invasion**		
Present	5	10.4
Absent	42	87.5
NA	1	2.1

*M: Male; F: Female; NA: Not available*.

Two experienced pathologists reviewed and classified histologic grades according to WHO criteria (El.Naggar et al., 2017). Histological differentiation was assigned as well-differentiated (grade I), moderately differentiated (grade II), and poorly differentiated (grade III) tumors. Microscopic examination of resected surgical margins was categorized according to the distance between the tumor and the surgical resection into negative (≥5 mm) and positive (<5 mm). Survival outcomes were evaluated as overall survival (OS), and the presence of desmoplasia, inflammatory infiltrate, vascular invasion, perineural invasion, and lymphatic invasion (Table 1) (El.Naggar et al., 2017). The study was approved by the Human Research Ethics Committee of A.C. Camargo Cancer Center (Number 2.481.465).

### Immunohistochemistry

Immunohistochemical assays were performed on 3-μm-thick sections of paraffin-embedded tissues as specified in Supplementary Table 1. Antigen retrieval was performed using an electric pressure cooker for 15 min. Endogenous peroxidase activity was suppressed using 6% H_2_O_2_ for 15 min before the sections were incubated with primary antibodies for 2 h. Immunohistochemical staining was performed with either the Advance (Dako, Hamburg, Germany) or Vectastain Elite ABC (Vector Laboratories, Burlingame, USA) kit, according to manufacturer's instructions. Slides were then exposed to diaminobenzidine tetrahydrochloride (Dako, Hamburg, Germany), and counterstained with Carazzi's hematoxylin.

### Immunohistochemical Analysis

The expression of CD3 +, CD4 +, CD8 +, CD20+, CD68+, CD83+, CD1a+, and PD-1+ cells were analyzed in the invasive margin (IM) and tumor center (TC). The methods for counting were adapted from published methods used to analyze TILs, dendritic cell, and PD-1 (Pellicioli et al., 2017; Zhou et al., 2018; Naruse et al., 2019; Ngamphaiboon et al., 2019). PD-L1+ cells were evaluated in the TC, where only membranous positivity was considered for scoring purposes. Complete circumferential or partial membranous staining of TC at any intensity was considered PD-L1-positive (+) and using reported parameters for the specific antibody clones (Tsao et al., 2017). OTSCC slides were screened at low power (100x), and then representative fields were selected at 200× magnification, as showed in Supplementary Figure 1. Images of each field were captured using an optical light microscope (Leica DFC450, Germany). Quantification of positive dendritic cells (CD83 and CD1a) and PD-1+ the individual value was the average of the total of 10 positive cell fields using ImageJ software (version 2.0). The density of CD3, CD4, CD8, CD20, and CD68 expression was scored using semiquantitative score based on the percentage of positive immune cells was scored as (1) negative (<5%), (2) weak (5–30%), (3) moderate (30–80%), and (4) strong (>80%) for each field (Ngamphaiboon et al., 2019), the individual scores are the mean values of the 10 fields. The results were shown as the mean number per slide. PD-L1 was considered positive in cases >1% and negative in cases <1% (Supplementary Table 2).

### Construction of Prognostic Score Based on Immune Characteristics

The determination of 2 groups of observations was accomplished by estimating a simple cut-point by using the maximum of the standardized log-rank statistic proposed by Lausen and Schumacher (Society, 2010). The optimal cut-points for cell surface markers were selected to enable sorting into low and high expression groups. The estimated cut-point was calculated for each marker, such as CD3+ 1.9 (low: 12; high: 26 patients); CD4+ 1.0 (low: 06; high: 42 patients); CD8+ 1.7 (low: 19; high: 29 patients); CD20+ 1.2 (low: 11; high: 37 patients); CD68+ 1.9 (low: 29; high: 19 patients); CD83+ 1.1 (low: 02; high: 46 patients); CD1a+ 5 (low: 09; high: 39 patients) and PD-1+ 6.6 (low: 16; high: 48 patients), (Supplementary Figure 2).

### Statistical Analysis

Analysis of the association between immune expression and patient demographic and clinicopathological characteristics was performed using Student's *t*-, Mann–Whitney *U*, Kruskal–Wallis, and Pearson's chi-square tests, as appropriate. The determination of two groups of observations for a simple cut-point was estimated by the use of the maximum of the standardized log-rank statistic proposed by Lausen and Schumacher (Society, 2010). Univariate overall survival (OS) probabilities were calculated using the Kaplan–Meier method. Comparisons among survival functions were performed with the log-rank test. The Cox semiparametric proportional hazards model was used to identify independent prognostic factors. Statistical analyses were performed using SPSS (version 23) and R (version 3.2.1) and the significance level was set at 5% for all statistical tests.

## Results

### Clinicopathological Features and Its Correlation With Survival

Forty-eight cases of OTSSC were retrieved. The clinicopathological features are presented in Table 1. Briefly, the mean age was 61.2 years (range 43–86 years), and males were represented (35 cases, 73%) more than females (13 cases, 27%). Twenty-eight (58.3%) patients had initial tumor stages (T1 and T2) and 20 (41.7%) had advanced-stage tumors (T3 and T4). Lymph node metastasis was observed in 18 patients (37.5%), and no patients presented distant metastasis. Initial clinical stages (Stages I and II) accounted for 23 cases (47.9%), while 25 (52.1%) presented with advanced clinical stage tumors (Stages III and IV). Seventeen patients (35.4%) developed recurrences, with a mean time to recurrence of 37.5 months (range: 0.1–97.2 months). The assessment of histological risk factors associated with prognosis is shown in Table 1. Interestingly, 16 patients (33.3%) had well-differentiated, 25 (52.1%) had moderately differentiated, and 7 (14.6%) had poorly differentiated tumors. Twenty tumors (41.6%) featured a desmoplastic stroma, and 40 displayed inflammatory infiltrates (83.3%). Desmoplasia was reported as weak in 9 cases (45%) and moderate in 7 patients (35%). Perineural invasion was reported in half of the surgical specimens (24 cases [50.0%]), while lymphovascular invasion was less frequent, observed only in 5 cases (10.4%) of patients, (Table 1).

The OS analysis showed an average estimate of 78.5 months (HR = 5.2, 95% CI 0.4–5.7). Univariate analysis showing the associations between clinicopathological features and OS are presented in Table 2. T3 stage (HR 29.6, 95% CI 3.0–295.3 (*p* = 0.04), N2 stage (HR 5.9, 95% CI 1.4–24.6 (*p* = 0.01), and poor histological grade (HR 12.7, 95% [CI] 1.4–117.1 (*p* = 0.02), were correlated with OS rates. Multivariate analysis revealed T stage (HR 9.172, 95% CI, 1.6–52.8) (*p* = 0.01) as a predictor of OS.

**Table 2:** Univariate and multivariate analysis for overall survival of oral squamous cell carcinoma patients.

	**HR**	**95.0% CI**		***P*-value**
**Overall survival**				
**Univariate model**				
**T stage**				
T1	1.0 (Ref.)			0.009
T2	3.867	0.348	42.972	0.271
T3	29.593	2.965	295.346	**0.004**
T4	3.872	0.228	65.766	0.349
**N stage**				
N0	1.0 (Ref.)			0.025
N1	0.608	0.070	5.266	0.652
N2	5.906	1.417	24.614	**0.015**
**Histological grade**				
Well	1.0 (Ref.)			0.027
Moderately	2.887	0.337	24.730	0.333
Poorly	12.770	1.393	117.061	**0.024**
**Overall survival**				
**Multivariate model**				
**T stage**	9.172	1.593	52.798	**0.013**
**CD3**	0.229	0.060	0.868	**0.030**

*Bold value is P < 0.05*.

### Characterization of the TIME Profile of OTSSC

**Figure 1:**
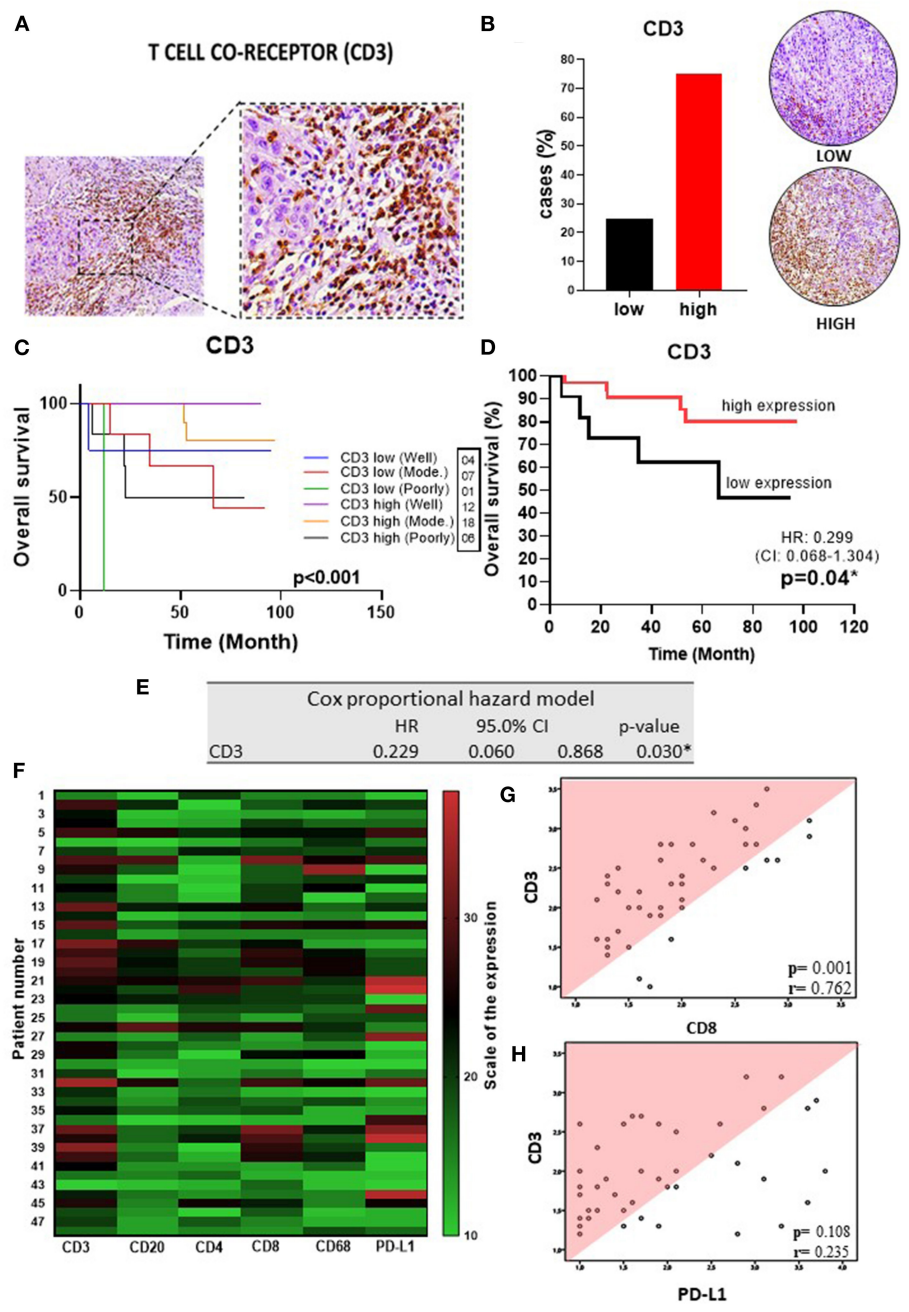
T cell co-receptor in OSSC **(A)** CD3 (left: 200X magnification; right panel: 400X magnification). **(B)** Representative images of OSCC tissues showing low and high CD3 expression, graph showing 36 cases (75.00%) with high CD3 tissues. **(C)** Well histological grade in CD3 high was associated with improved OS (*p* < 0.001). **(D)** High CD3 expression was associated with improved OS (*P* = 0.04). **(E)** Table showing multivariate analysis for CD3 (*p* = 0.03). **(F)** Heat map showing TILs, slim and PD-L1 expressions in OSSC. **(G)** Correlation of the CD3 rate with expression patterns of CD8 (*r* = 0.76; *p* = < 0.001) and **(H)** PD-L1 (*r* = 0.411; *p* = 0.004) by Spearman rank correlation coefficient. CI, confidence interval; HR, hazard ratio; OS, overall survival; OSCC, oral squamous cell carcinoma; CD3, T cell co-receptor; Mod, Moderately.

**Figure 2:**
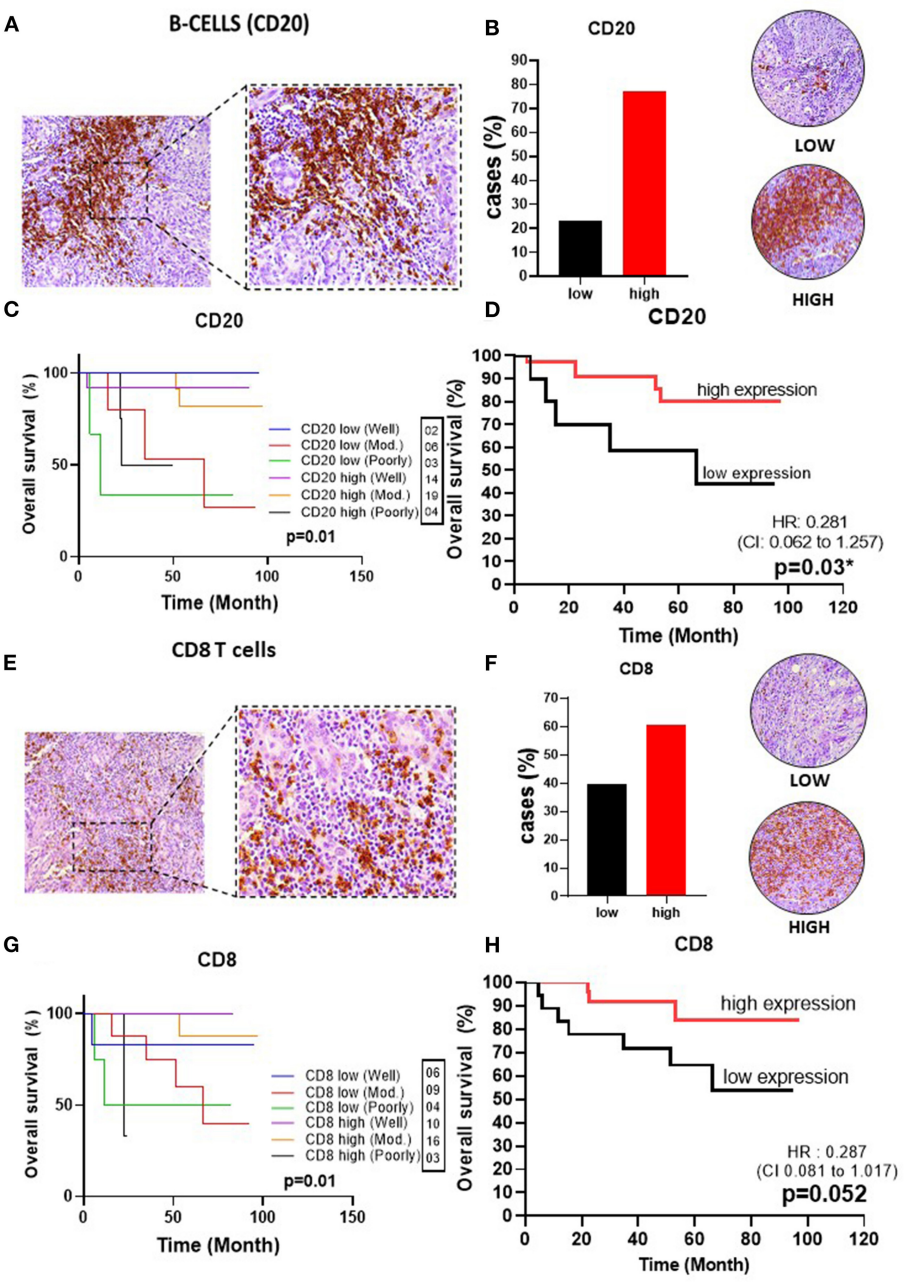
Infiltrating lymphocytes in OSSC. **(A)** CD20 (left: 200X magnification; right panel: 400X magnification). **(B)** Representative images of OSCC tissues showing low and high expressions of CD20, graph showing 37 cases (77.08%) with high CD4 tissues. **(C)** Well histological grade in CD20 high was associated with improved OS (*p* = 0.01). **(D)** High CD20 expression was associated with improved OS (*p* = 0.03). **(E)** CD8 in OSCC (left: magnification 200X; right panel: magnification 400X). **(F)** Representative images of OSCC tissues showing low and high CD8 expressions, graph showing 29 cases (60.41%) with low CD68 **(G)** Well histological grade in CD8 high was associated with improved OS (*p* = 0.01); **(H)** High CD8 expression was associated with improved OS (*P* = 0.052). CI, confidence interval; HR, hazard ratio; OS, overall survival; OSCC, oral squamous cell carcinoma; CD20, B cell; CD8, cytotoxic T cell; Mod, Moderately.

To delineate the OTSCC TIME profile, stratification was based on individualized cutoff values for the optimized prognostic power of each marker (Figures 1A, 2AE, 3AE, 4AE, 5AD). All patients were classified into low or high expression groups for each marker, and the percentages of individuals in each group were compared.

**Figure 3:**
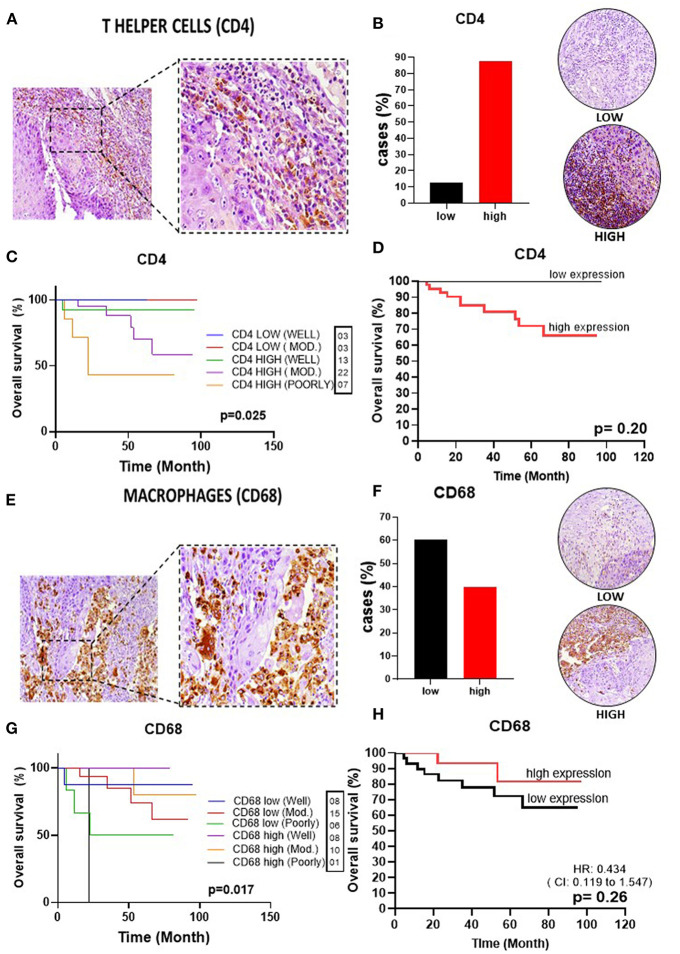
Infiltrating T helper cells and macrophages in OSSC. **(A)** CD4 (left: 200X magnification; right panel: 400X magnification). **(B)** Representative images of OSCC tissues showing low and high CD4 expression, graph showing 42 cases (87.5%) of high CD4 tissues. **(C)** Well histological grade in CD4 low was associated with improved OS (*p* = 0.02). **(D)** High expression was unrelated to OS (*P* = 0.21). **(E)** CD68 in OSCC (left: magnification 200X; right panel: magnification 400X). **(F)** Representative images of OSCC tissues showing low and high expression, graph showing 29 cases (60.42%) with low CD68 low 60.42%; **(G)** Well histological grade in CD68 high was associated with improved OS (*p* = 0.017). **(H)** High CD68 expression was unrelated to OS (*P* = 0.18). CI, confidence interval; HR, hazard ratio; OS, overall survival; OSCC, oral squamous cell carcinoma; CD4, T helper cell; CD68, pan-macrophage; Mod, Moderately.

**Figure 4:**
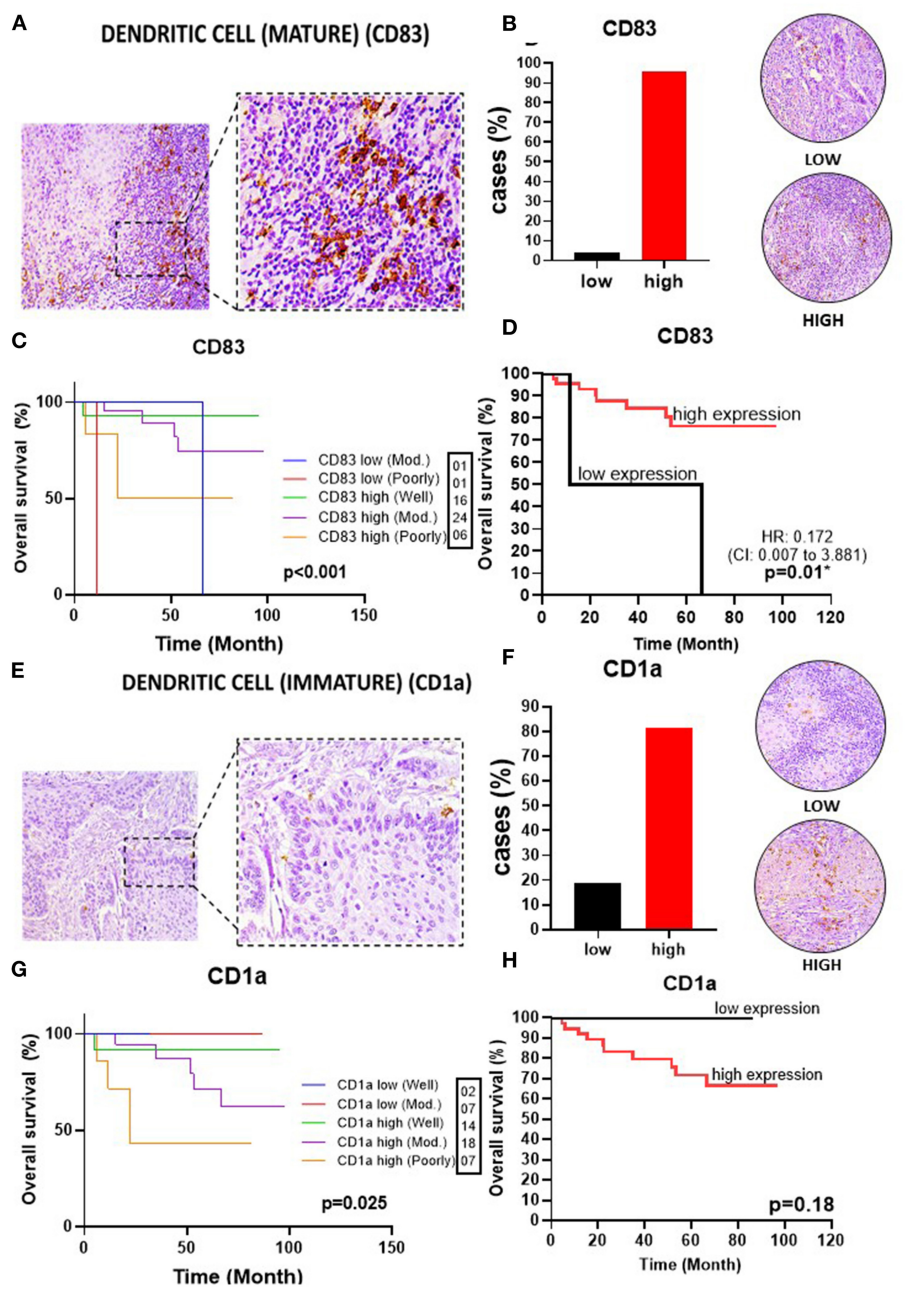
Infiltrating dendritic cells in OSSC. **(A)** CD83 (left: 200X magnification; right panel: 400X magnification). **(B)** Representative images of OSCC tissues showing low and high CD83 expression, graph showing 46 cases (95.83%) with high CD83 tissues. **(C)** Well histological grade in CD83 high was associated with improved OS (*p* < 0.001). **(D)** High CD83 expression was associated with better OS (*P* = 0.01). **(E)** CD1a in OSCC (left: magnification 200X; right panel: magnification 400X). **(F)** Representative images of OSCC tissues showing low and high CD1a expression, graph showing 39 (81.25%) cases with high CD1a. **(G)** Well histological grade in CD1a low was associated with improved OS (*p* = 0.025). **(H)** High CD1a expression did not impact OS (*P* = 0.18). CI, confidence interval; HR, hazard ratio; OS, overall survival OSCC, oral squamous cell carcinoma; CD83, mature dendritic cell; CD1a, immature dendritic cell; Mod, Moderately.

**Figure 5:**
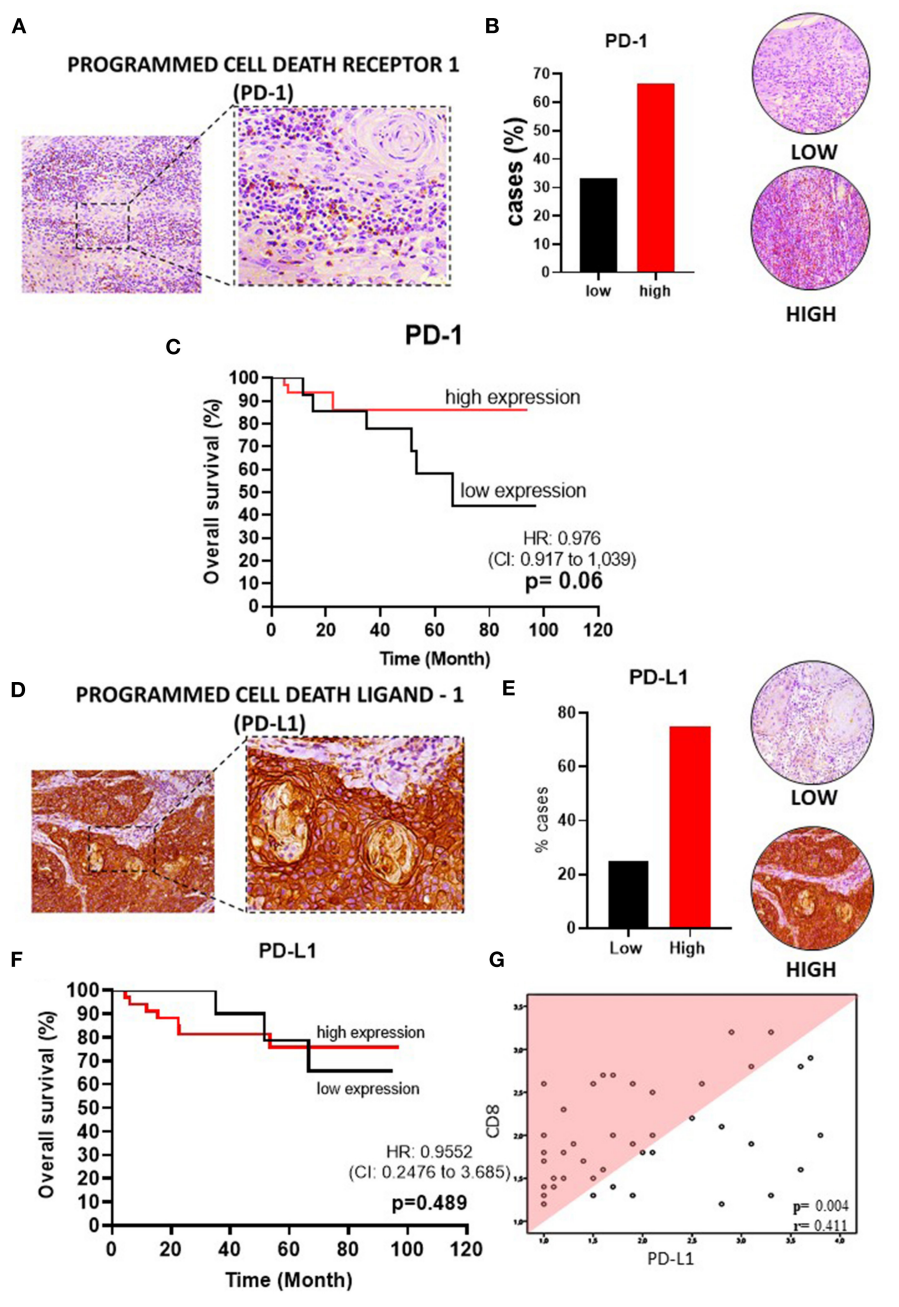
PD-1 and PD-L1 in OSSC. **(A)** PD-1 in OSCC (left: 200X magnification; right panel: 400X magnification). **(B)** Representative images of OSCC tissues showing low and high PD-1 expression, graph showing 32 cases (66.67%) of high PD-1. **(C)** High PD-1 expression was not associated with OS (*P* = 0.06). **(D)** PD-LD-1 in OSCC (left: 200X magnification; right panel: 400X magnification). **(E)** Representative images of OSCC tissues showing low and high PDL-1 expression, graph showing 36 cases (75%) with high PD-L1. **(F)** High PD-L1 expression was not associated with OS. **(G)** Correlation of CD8+ rate with expression patterns of PD-L1 by Spearman rank correlation coefficient (*r* = 0.480; *p* = 0.001). CI, confidence interval; HR, hazard ratio; OS, overall survival; OSCC, oral squamous cell carcinoma, PD-1, programmed cell death receptor-1; PD-L1, programmed cell death ligand-1; Mod, Moderately.

Upon analysis of the percentage of tumors demonstrating high vs. low lymphocyte subpopulation infiltration, we observed a major increase in the high expression groups for CD3^+^ (75.0%) (Figure 1B), CD20^+^ (77.1%) (Figure 2B), CD8^+^ (60.4%) (Figure 2F), and CD4^+^ (87.5%) cells (Figure 3B). In contrast, the majority of tumors expressed low frequencies of CD68+ macrophages (Figure 3F). Remarkably, almost all OTSCC presented high expression profiles of mature dendritic cells, evaluated by CD83 expression (95.8%) (Figure 4B), as well as, immature dendritic cells assessed through CD1a expression (81.3%) (Figure 4F). In addition to the infiltrating cell populations, we also evaluated the expression of the immune checkpoint molecules PD-1 and PD-L1 due to their immunosuppressive activity. Both proteins were highly expressed in most of the samples with PD-1 in 66.7% (Figure 5B) and PD-L1 in 75.0% (Figure 5E).

To better understand the OTSCC immunoregulatory networks established in the TIME, we analyzed the correlation between the expression of the evaluated markers. Using the original data of the quantification of TILs and PD-L1 markers, a heatmap was constructed showing the relative expression levels for each marker across all patient samples (Figure 1F). Performing a Spearman correlation analysis, we observed a clear correlation between CD3+ cells and CD8+ cells (*p* < 0.05, *R* = 0.76) (Figure 1G), as well as with PD-L1+ cells (*p* < 0.05, *R* = 0.41) (Figure 1H). In addition, the densities of CD8+ cells significantly correlated with the levels of CD3+, CD20+, CD68+ subsets with *R* values 0.76; 0.71; and 0.59 (Spearman correlation analysis, *p*-values < 0.05) (Supplementary Table 3); and PD-L1+ cells (Figure 5G).

### Increased Expression of Infiltrating T and B Lymphocytes Are Correlated With Improved Survival in OTSCC

We next explored whether the expression of the immune markers influenced OS. These analyses showed that infiltrating CD3+ T cells were correlated with an improved OS (*p* = 0.04) (HR 0.3, 95%CI, 0.1–1.3) (Figure 1D). Also, statistically significant variables identified by univariate analysis were evaluated by using the multivariate Cox proportional regression analysis hazard model. Not surprisingly, CD3^+^ T cells were identified as an independent prognostic factor (*p* = 0.03) (HR 0.1, 95%CI, 0.1–0.9), supporting the prognostic value of CD3 markers in OTSCC (Figure 1E).

Analysis of CD8+ T cells revealed that higher levels were also associated with better OS (HR 0.3, 95%CI, 0.1–1.1), with a *p*-value of 0.052 (Figure 2H). CD4+ T cells, which play an important role in immunoregulation, were not correlated with OS (*p* = 0.21) (Figure 3D). Lastly, we found that high CD20+ B cells were correlated with an improved OS (*p* = 0.03) (HR 0.3, 95% CI, 0.1–1.3) (Figure 2D).

### High Expression of Mature Dendritic Cells in the TME Are Correlated With Higher Survival

Given the importance of myeloid-derived immune effector cell subpopulations in the regulation of the TIME, we evaluated the pan-macrophage immunomarker CD68 to assess the presence of macrophages (both M1 and M2). We did not observe a correlation between expression of CD68^+^ cells and OS (HR 0.4, 95%CI, 0.1–1.6) (*p* = 0.27) (Figure 3H). In addition, we also evaluated the presence of dendritic cells, due to their important role in T cell activation and immunoregulation. Our results demonstrated high prevalence of mature dendritic cells (CD83^+^) in the TME was correlated with an improved OS (HR 0.2, 95%CI 0.0–3.4) (*p* = 0.01) (Figure 4D). In contrast, immature dendritic cells did not impact survival (*p* = 0.18) (Figure 4H).

### TIME Correlates With Histological Grade and Survival in OTSCC

We stratified patients by histological grade (well, moderaty, and poorly differentiated) with the expression of the markers CD3, CD4, CD8, CD20, CD68, CD83, CD1a, and correlating with OS.

This analysis demonstrated a strong association of CD3+ and CD8+ T cells with a histological grade of well-differentiated and high expression of T lymphocytes with an improved OS (*p* < 0.001 and *p* = 0.001, respectively) (Figures 1C, 2G). CD4+ T lymphocytes showed the opposite where patients with low expression show improvement in OS, but in this group no patient was found with poorly differentiated histological grade (*p* = 0.025) (Figure 3C). CD20+ B cells were correlated with OS improvement when they have a well-differentiated histological grade (*p* = 0.01) (Figure 2C).

CD68+ pan-macrophage cells showed the pattern of well histological differentiation and correlated with OS improvement (*p* = 0.017) (Figure 3G). CD83+ mature dendritic cells showed the pattern of high expression with improved survival when compared to low expression, independent of the degree of differentiation between these two groups (*p* < 0.001) (Figure 4C). Non-low expression of the immature CD1a dendritic cell marker correlated with improved OS (*p* = 0.025) (Figure 4G).

### TME Expression of PD-1 and PD-L1 Were not Associated With Survival Outcomes

The immune checkpoint molecules PD-1 and PD-L1 can suppress immunity, thereby preventing autoimmunity and protecting tissues from immune-mediated injury. However, in the context of cancer, they can inhibit beneficial anti-tumor immune responses. Although most of the tumors displayed high expression of both PD-1 (HR 1.0, 95% CI, 0.9–1.0, *p* = 0.06) and PD-L1 (HR 1.0, 95% CI, 0.3–3.7, *p* = 0.48), the expressions of these proteins in the TME and tumor center were not correlated with OS (Figures 5C,F).

To gain further insights into immunoregulatory networks in the TME, we performed a correlation matrix between all immune cell subpopulations and checkpoint inhibitor molecules. We observed a significant positive correlation (*p* < 0.05) between the densities of PD-1+ cells and cell subpopulations expressing CD3+, CD4+, CD8+, CD20+, and CD83+. In addition, a strong positive correlation was seen between CD20+ B cells and T cell subpopulations (CD3+, CD4+ and CD8+), which could be indicative of tertiary lymphoid structures (Supplementary Table 2).

## Discussion

Cancer biology research focused on the TIME has grown exponentially in recent years. Accumulating evidence suggests that OSCC is an immunosuppressive disease, and consequently immunotherapy has emerged as a novel approach to restore anti-tumor responses and to overcome escape mechanisms utilized by tumor cells (Ferris, 2015; Moskovitz and Ferris, 2018; Galvis et al., 2020). Nevertheless, it is important to recognize that the immune system is extremely complex, and that environmental factors such as tobacco and alcohol use can modify the antitumor immune response (de la Iglesia et al., 2020). Therefore, there is an acute need to recognize TIME profiles and assess their impacts on OSCC survival.

Herein we demonstrate that OTSCC in patients with tobacco and alcohol exposures is characterized by high levels of total T cells (CD3+), CD8+ T cells, and CD4+ T cells, as well as B lymphocytes (CD20+), and immature (CD1a) and mature (CD83) dendritic cells. Moreover, tumor cells showed a high expression of the immune checkpoint molecule PD-L1, while surrounding immune cells exhibited high PD-1 expression. In contrast, the TME presented low levels of macrophages (CD68). The influence of tumor immune infiltrate on survival curves showed particular promise for the CD3+ T cell co-receptor, CD20+ B cells and CD8+ T cells as predictors of survival, and identified CD3 as an independent prognostic marker.

The immune microenvironment is composed primarily of TILs (T and B lymphocytes and NK cells), macrophages, and dendritic cells (Munn and Bronte, 2016; Hadler-Olsen and Wirsing, 2019). All components play critical roles in the detection and elimination of tumor cells, thereby contributing to arresting tumor progression and proliferation (Kim, 2007; Ferris, 2015). The activation of T cells requires the T-cell receptor complex, which includes the co-receptor CD3 (Perri et al., 2020). Our findings showed that OTSCC featured a high expression of CD3+ T cells that was significantly correlated with improved survival and acts as an independent prognostic indicator. Past studies have shown that the presence of CD3+ T cells in the TME is related to improved prognosis in a wide range of tumors (Zhou et al., 2018, 2020). Supporting our findings, recent studies have reported the abundance of CD3+ T cells in the TME as an independent prognostic factor for OSCC, improving OS and recurrence-free survival (Zhou et al., 2018, 2020). Thus, the high expression of TME CD3+ cells is likely reflecting immunocompetent T lymphocytes with anti-tumor activity, and also highlights its potential roles as an accurate prognostic marker and for the selection of patient candidates for therapies targeting T lymphocytes (Almangush et al., 2020).

B lymphocytes play an important role in antigen presentation and antibody production in the early stages of tumor development (Quan et al., 2016; Taghavi et al., 2018; Hadler-Olsen and Wirsing, 2019). Our data correlated a high expression of CD20 with improved OS. Despite the importance of B cells in antitumor activity, there remains a paucity of evidence regarding their prognostic role. Our finding that TME CD20+ B cells are strongly correlated with T cells could be reflecting the formation of tertiary lymphoid structures, which have been associated with improved survival in OSCC (Wirsing et al., 2014). Notably, our results reinforce the importance of understanding the role of B cells in the TIME and their role as a prognostic factor.

Immune activation is driven through interactions between immune cells and tumor-associated antigens (Perri et al., 2020). CD8+ cytotoxic T cell activation follows interaction with antigen-presenting cells. Our data revealed that a high prevalence of T cells (likely including cytotoxic CD8+ T cells) in the invasive margin tends to be associated with higher OS. The presence of cytotoxic T cells in the TME has been highlighted as a key-point in the interactions between tumor and immune cells (Ferris, 2015; Hadler-Olsen and Wirsing, 2019; Perri et al., 2020). Our findings are consistent with previous studies showing that the high expression of CD8+ cytotoxic T cells is correlated with improved survival in OTSSC (Nguyen et al., 2016; Zhou et al., 2018; Shimizu et al., 2019). Due to all evidence and the biological role of CD8+ T cells in the TME, CD8 may prove to be a fundamental molecule to assess as a prognostic marker for OTSSC patients.

CD4+ T helper cells act as regulators of the immune response, thus avoiding exacerbated immune responses, particularly those mediated by cytotoxic T cells (Perri et al., 2020). Treg cells represent a minor heterogenic subsite of CD4+ T cells with the potential to induce suppression of CD8+ T cells and may contribute to carcinogenesis and cancer progression by two mechanisms; first, by limiting immune function upon tumor recognition; and second, by suppressing the cellular activity of cytotoxic T cells (Dayan et al., 2012; Hadler-Olsen and Wirsing, 2019; Perri et al., 2020). Our results demonstrated that patients with increased CD4+ expression tended to have a worse prognosis, although statistical significance was not established. Previous studies evaluating the impact of CD4+ T cells also did not reach statistical significance with respect to their influence on OS. These data indicate the antagonistic effect of some T cell subpopulations in the TME, and demonstrate the importance of characterization of specific cell subpopulations in the TIME, mainly Treg cells, which often express Foxp3, a marker not included in our study (Perri et al., 2020).

Tumor-associated macrophages present two different phenotypes: M1, which exerts an antitumor role; and M2, which exerts a protumor immunosuppressive role (Alves et al., 2018). CD68 is a pan-macrophage marker, which has been related to tumor-suppressor activity (Alves et al., 2018; Hadler-Olsen and Wirsing, 2019). We did not observe statistical differences between CD68+ cells and survival outcomes. A recent meta-analysis found no significant association between the CD68 pan-macrophage marker and OS (Hadler-Olsen and Wirsing, 2019). Moreover, a recent literature review noted the difficulty in evaluating this marker due to data variability and study methodologies (Alves et al., 2018).

Dendritic cells (DC) are responsible for recognizing and presenting antigens to immune cells, especially T cells (Hansen and Andersen, 2017; Perri et al., 2020). Currently, there are a large number of markers for characterizing DC subpopulations (Pellicioli et al., 2017; Jardim et al., 2018). In our study, we selected CD83 (mature DCs) and CD1a (immature DCs) because of their well-established biological properties and previous investigations (Gomes et al., 2016; Pellicioli et al., 2017; Jardim et al., 2018). Our findings showed that OTSCC patients presented high expression of CD83+ DCs, and that they were significantly correlated with improved survival. Pellicioli *et al*. showed that the presence of mature DCs is associated with the early stages of OTSSC development (Pellicioli et al., 2017). In contrast, Jardim et al., did not find a correlation between CD83 and OS (Jardim et al., 2018).

We used CD1a expression as an indicator of immature DCs. CD1a is involved in antigen presentation by dendritic Langerhans cells to T lymphocytes (Gondak et al., 2012; Jardim et al., 2018). Our data demonstrated that increased levels of CD1a+ cells tended to result in a worse prognosis, although statistical significance was not established. However, Jardim et al. showed that lower levels of immature peritumoral DCs predict higher recurrence rates and shorter survival (Jardim et al., 2018). The presence of immature DCs often demonstrates that the immune system is in the stage of maturation and recruitment, and thus predicts worse survival. The opposite effect is suggested by the presence of mature DCs, which reflects the presence of active T lymphocytes, and predicts an active immune response and improved prognosis (Hansen and Andersen, 2017; Jardim et al., 2018).

T cells express the immunological checkpoint protein PD-1, which interacts with its receptor on neoplastic and immune cells, PD-L1, leading to the inhibition of cytolytic antitumor responses, thereby promoting immunologic escape of tumor cells (Ferris, 2015; Gato-Cañas et al., 2017; Gong et al., 2018; Moskovitz and Ferris, 2018). In our study, levels of PD-1 did not correlate with prognosis in OTSSC. Thus, our study demonstrates the presence of an active microenvironment that drives the expression of immune checkpoint inhibitors; however, this microenvironment manages to remain active through the recruitment of CD8+ T cells, reinforcing the potential use of immune checkpoint inhibitor therapies (Ferris, 2015; Gato-Cañas et al., 2017; Kansy et al., 2017; Gong et al., 2018).

The presence of PD-L1 in tumor cells indicates an active mechanism of immune escape (Ferris, 2015; Moskovitz and Ferris, 2018; Perri et al., 2020). In our study, there was no clear effect of PD-L1 expression on OS. The expression of PD-L1 by tumor cells can facilitate immunologic escape by inhibiting cytotoxic T cell function; therefore, increased PD-L1 can correlate with a worse prognosis. On the other hand, the expression of PD-L1 by tumor cells may be secondary to the production of IFN-gamma by infiltrating T cells, which is often associated with better outcomes (Moskovitz and Ferris, 2018; Perri et al., 2020). Previous studies have associated PD-L1 overexpression with reduced survival (Lin et al., 2015; De Vicente et al., 2019; Lenouvel et al., 2020) others show the opposite, where prognostic significance is not established (Huang et al., 2020). These conflicting results show the importance of the need for standardization of marking for this protein. A recent study has demonstrated several PD-L1 marker patterns in OTSCC, which associated four patterns with distinct biological processes (genetic modifications and adaptive immune resistance, immune ignorance, intrinsic induction, and immune tolerance), and demonstrated a change in survival when evaluating the different patterns (Miranda-Galvis et al., 2020). Thus, the study shows the potential prognostic value of PD-L1, however studies evaluating these patterns in different cohorts and tumors will clarify their potential as broadly used markers.

Another finding observed in our study was the stratification of the patients regarding the histological grade. The histological grade is a known prognostic factor in OTSSC, and it has been described that higher grades of TILs or increasing CD3+ or CD8 + cell densities are associated with histological grades (Giraldo et al., 2019; Almangush et al., 2020; Zhou et al., 2020). Our study observed that the presence of TIME influences survival of the patients even when stratifying the patients by the histological grade. Importantly, CD3+ T cells remained an independent marker.

Our studies limitations are; First, the strict inclusion criteria that aimed to study a more homogeneous population, defined by tobacco and alcohol consumption, led to a smaller sample size, Second, the cell population of macrophages was evaluated by using the pan-macrophage antibody, which did not allow the identification of M1 and M2 subtypes, Third, the assessment of immune cell profiles through immunohistochemistry only allows the identification of the presence of the cells, but does not address their specific immune functional activity, and Fourth, due to the sample size stratification by histological grade led to small sub-group sizes, limiting the ability to generalize these particular findings.

In summary, we provide evidence that OTSCC patients who smoke tobacco and drink alcohol have an active TIME, which is associated with better OS. We identified specific TIL subpopulations, including CD3+ T cells, CD20+ B cells, and CD83+ mature dendritic cells, that are associated with better survival rates. Moreover, increased expression of CD3+ T cells was identified as an independent prognostic marker, suggesting a potential biomarker for therapeutic targets in OTSCC.

## Data Availability Statement

The raw data supporting the conclusions of this article will be made available by the authors, without undue reservation.

## Ethics Statement

The studies involving human participants were reviewed and approved by Human Research Ethics Committee of A.C. Camargo Cancer Center. The patients/participants provided their written informed consent to participate in this study.

## Author Contributions

RS: study concepts, study design, data acquisition, data analysis and interpretation, statistical analysis, manuscript preparation, and manuscript editing. MM: data acquisition, data analysis and interpretation, statistical analysis, and manuscript preparation. BM: data analysis and interpretation, and manuscript preparation. AL: data interpretation and manuscript review. LS: data acquisition and quality control. OA: data acquisition and quality control, and manuscript review. AS-S: data analysis and interpretation, and manuscript review. CP: data acquisition and quality control. PV: study concepts, study design, data analysis and interpretation, and manuscript review. KG: study concepts, study design, data analysis and interpretation, and manuscript review. LK: study concepts, study design, data quality control and algorithm, data analysis and interpretation, and manuscript review. All authors contributed to the article and approved the submitted version.

## Conflict of Interest

The authors declare that the research was conducted in the absence of any commercial or financial relationships that could be construed as a potential conflict of interest.
