# Supplementary material for: Increased Tumor Immune Microenvironment CD3+ and CD20+ Lymphocytes Predict a Better Prognosis in Oral Tongue Squamous Cell Carcinoma
Source: Front Cell Dev Biol. 2021 Feb 18;8:622161. doi: 10.3389/fcell.2020.622161 (PMC7951138; doi:10.3389/fcell.2020.622161)
Supplement: Supplementary file 1 [file Table_1.DOCX]

Supplementary Material

**Supplementary Table 1**. Antibodies and specifications.


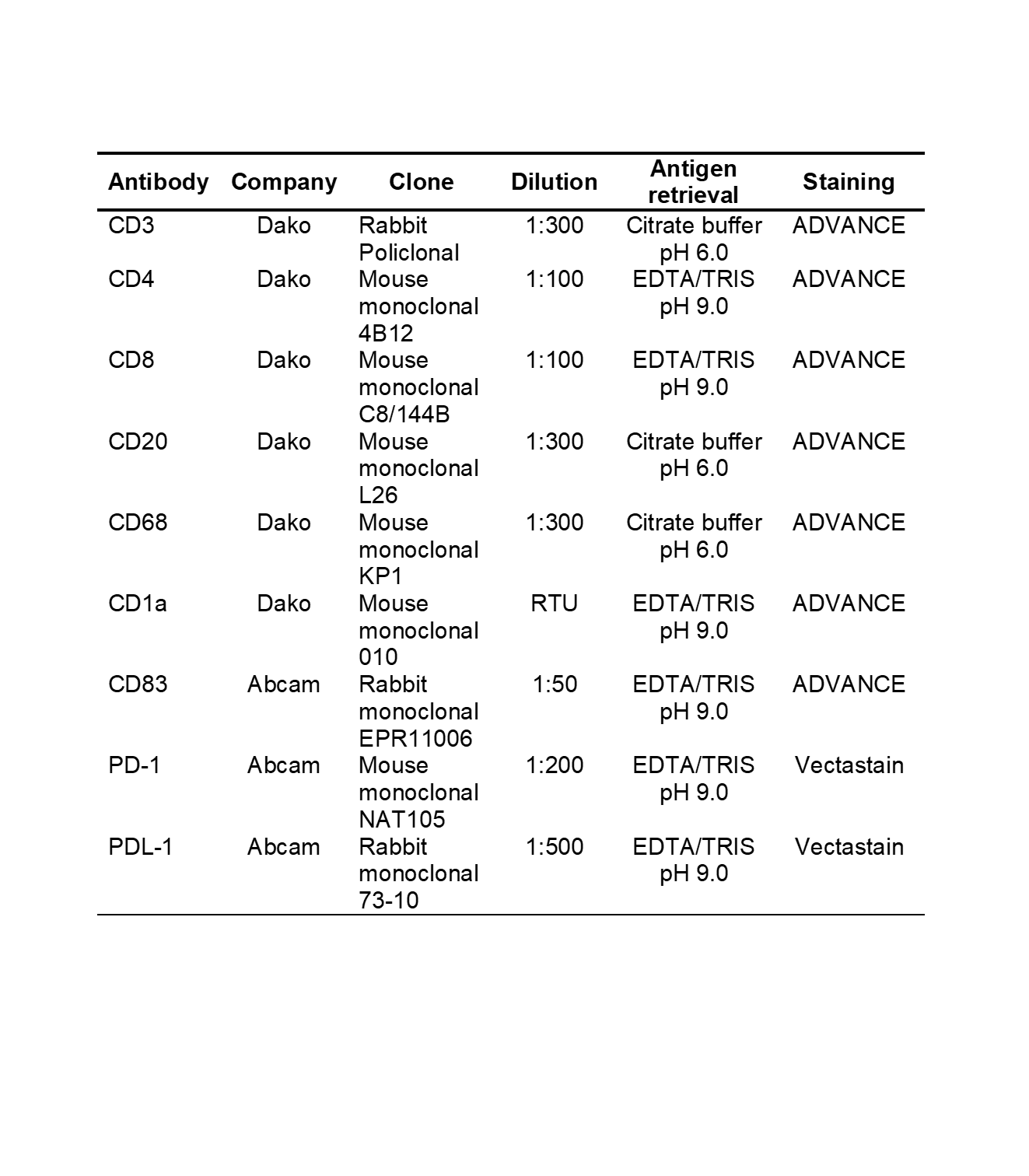


**Supplementary Figure 1.** Immunohistochemical panel used in our study. Representative images showing positivity for **(A)** CD3; **(B)** CD4; **(C)** CD8; **(D)** CD20; **(E)** CD68; **(F)** CD83; **(G)** CD1a; **(H)** PD-1 and **(I)** PD-L1. Original magnification 200X


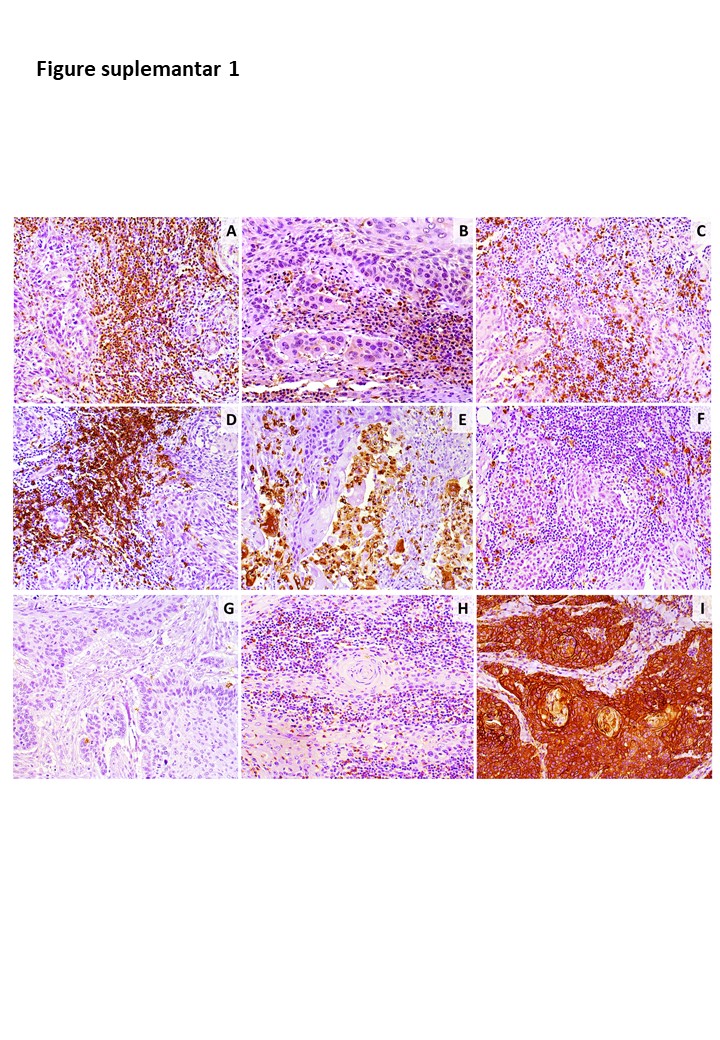


**Supplementary Figure 2. (A)** CD3 Standardized log-rank graph showing low and high expression based on a simple cut-point (1. 9). **(B)** CD20 Standardized log-rank graph showing low and high expression based on a simple cut-point (1.2). **(C)** CD8 Standardized log-rank graph showing low and high expression based on a simple cut-point (1.7) . **(D)** CD4 Standardized log-rank graph showing low and high expression based on a simple cut-point (1.0). **(E)** CD68 Standardized log-rank graph showing low and high expression based on a simple cut-point (1.9). **(F)** CD83 Standardized log-rank graph showing low and high expression based on a simple cut-point (1.1). **(G)** CD1a Standardized log-rank graph showing low and high expression based on a simple cut-point (5). **(H)** PD-1 Standardized log-rank graph showing low and high expression based on a simple cut-point (6.6).


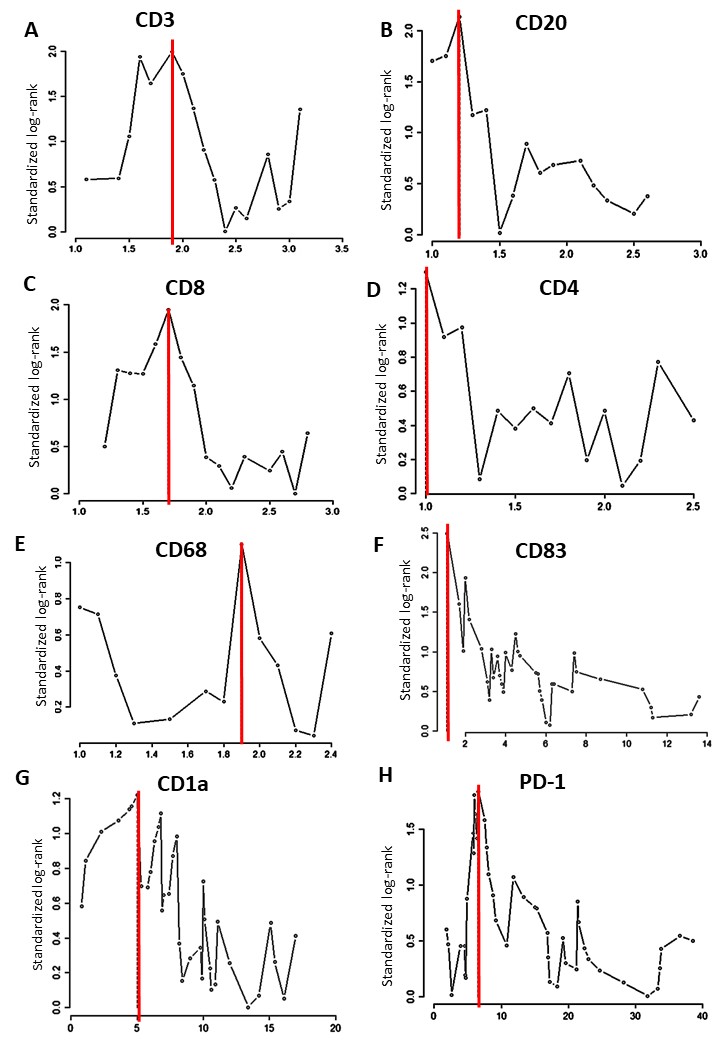


**Supplementary Table 2**. Spearman’s correlation density of cells between immune markers. The first row shows the Spearman’s correlation r^2^ value and the second row the p value for each marker

|  | **CD3** | **CD4** | **CD8** | **CD20** | **CD68** | **CD83** | **CD1a** | **PD-1** | **PD-L1** |
| --- | --- | --- | --- | --- | --- | --- | --- | --- | --- |
| **CD3** |  | 0.199 | 0.762^**^ | 0.788^**^ | 0.585^**^ | 0.145 | -0.093 | 0.316^*^ | 0.235 |
|  |  | 0.175 | 0.001 | 0.001 | 0.001 | 0.324 | 0.530 | 0.029 | 0.108 |
| **CD4** |  |  | 0.301^*^ | 0.428^**^ | 0.109 | 0.211 | -0.079 | 0.623^**^ | 0.388^**^ |
|  |  |  | 0.037 | 0.002 | 0.459 | 0.149 | 0.592 | 0.000 | 0.006 |
| **CD8** |  |  |  | 0.711^**^ | 0.592^**^ | 0.228 | -0.174 | 0.472^**^ | 0.411^**^ |
|  |  |  |  | 0.000 | 0.001 | 0.119 | 0.237 | 0.001 | 0.004 |
| **CD20** |  |  |  |  | 0.556^**^ | 0.239 | -0.214 | 0.471^**^ | 0.237 |
|  |  |  |  |  | 0.001 | 0.102 | 0.144 | 0.001 | 0.105 |
| **CD68** |  |  |  |  |  | 0.050 | -0.334^*^ | 0.258 | 0.316^*^ |
|  |  |  |  |  |  | 0.733 | 0.020 | 0.077 | 0.029 |
| **CD83** |  |  |  |  |  |  | -0.296^*^ | 0.348^*^ | 0.243 |
|  |  |  |  |  |  |  | 0.041 | 0.015 | 0.095 |
| **CD1a** |  |  |  |  |  |  |  | -0.060 | -0.218 |
|  |  |  |  |  |  |  |  | 0.686 | 0.137 |
| **PD-1** |  |  |  |  |  |  |  |  | 0.480^**^ |
|  |  |  |  |  |  |  |  |  | 0.001 |
| **PD-L1** |  |  |  |  |  |  |  |  |  |

*Correlation significant at 0.05 (bilateral)

** Correlation significant at 0.01 (bilateral)

**Supplementary Table 3. Cell counts data for each marker**

| Patient | CD3* | CD4* | CD8* | CD20* | CD68* | CD83** | CD1a** | PD-1** | PD-L1*** |
| --- | --- | --- | --- | --- | --- | --- | --- | --- | --- |
| 1 | 1.5 | 2 | 1.5 | 1.1 | 1.5 | 1.1 | 20.3 | 6.6 | 1 |
| 2 | 2.8 | 1 | 1.8 | 2.1 | 2.2 | 6.4 | 5.8 | 15.3 | 2 |
| 3 | 2.3 | 1.2 | 1.3 | 1.1 | 1.2 | 3.1 | 15.4 | 8.8 | 2 |
| 4 | 2.4 | 1.3 | 2 | 1.2 | 1.7 | 11.3 | 5 | 15 | 2 |
| 5 | 2.8 | 2.1 | 2.1 | 2.6 | 2.6 | 3.8 | 6.6 | 33.8 | 2 |
| 6 | 1.1 | 1.2 | 1.6 | 1 | 2.1 | 5.6 | 0.8 | 2.1 | 2 |
| 7 | 2 | 2.2 | 2 | 1.5 | 1.9 | 4 | 6 | 23 | 2 |
| 8 | 2.9 | 1.2 | 3.2 | 2.9 | 2.5 | 4.5 | 5.3 | 1.8 | 2 |
| 9 | 2.6 | 1 | 1.8 | 1.8 | 3.4 | 2.2 | 7.4 | 4.7 | 1 |
| 10 | 2 | 1.1 | 1.8 | 1 | 2 | 2.8 | 17.9 | 10.8 | 2 |
| 11 | 2.4 | 1 | 2 | 1.5 | 2.4 | 3.7 | 10.5 | 8.1 | 1 |
| 12 | 2.1 | 1.1 | 2 | 1.5 | 1 | 13.2 | 17 | 22.4 | 2 |
| 13 | 3.1 | 2.3 | 2.5 | 2.2 | 1.7 | 7.4 | 10 | 21.4 | 2 |
| 14 | 2.2 | 1.3 | 1.4 | 1.1 | 1.5 | 3.4 | 7.7 | 1.3 | 1 |
| 15 | 3 | 2.1 | 2.6 | 1.8 | 2.5 | 2.2 | 15.1 | 13.3 | 2 |
| 16 | 2 | 1.5 | 1.5 | 1.3 | 1.5 | 4.3 | 12 | 9.2 | 2 |
| 17 | 3.2 | 2 | 2.3 | 2.7 | 1.3 | 6 | 10.9 | 19.6 | 2 |
| 18 | 2.8 | 1.7 | 2.7 | 2.2 | 2.3 | 8.7 | 4.4 | 24.7 | 2 |
| 19 | 3 | 2 | 2.6 | 2.3 | 2.5 | 3.3 | 11.1 | 36.6 | 2 |
| 20 | 2.8 | 2 | 1.9 | 2.2 | 2.5 | 16.7 | 5.3 | 28.2 | 2 |
| 21 | 2.6 | 2.6 | 2.8 | 2.5 | 2.1 | 13.6 | 7 | 31.8 | 2 |
| 22 | 2.3 | 2.8 | 2 | 1.9 | 1.9 | 6 | 4.6 | 38.5 | 2 |
| 23 | 2.4 | 2.1 | 2 | 2.2 | 2 | 3.9 | 2.3 | 45.4 | 1 |
| 24 | 1.6 | 1.9 | 1.9 | 1.4 | 1.7 | 6.2 | 2.3 | 33.6 | 2 |
| 25 | 1.6 | 1.7 | 1.3 | 1.1 | 1.9 | 2 | 9.8 | 4.5 | 2 |
| 26 | 2.5 | 2.6 | 2.6 | 3 | 2.1 | 3.4 | 8.4 | 33.3 | 2 |
| 27 | 1.5 | 1.8 | 1.3 | 1.3 | 1.9 | 6.2 | 14.2 | 21.2 | 2 |
| 28 | 2.5 | 1.5 | 1.4 | 1.8 | 1.5 | 3.6 | 21.2 | 6 | 1 |
| 29 | 2.5 | 1 | 2.3 | 1.6 | 2.4 | 11.2 | 6.3 | 4.6 | 2 |
| 30 | 1.4 | 1.3 | 1.3 | 1.2 | 1 | 14.8 | 6.9 | 5.8 | 1 |
| 31 | 2 | 1.3 | 1.6 | 1 | 1.2 | 4 | 15.1 | 19.2 | 2 |
| 32 | 3.5 | 1.7 | 2.8 | 2.6 | 2.5 | 13.9 | 9 | 17.2 | 2 |
| 33 | 2.1 | 1.6 | 1.2 | 1.3 | 1.1 | 5.8 | 10.6 | 5.9 | 1 |
| 34 | 1.9 | 1.9 | 1.7 | 1.4 | 1.9 | 6.3 | 8.2 | 11.8 | 2 |
| 35 | 2.3 | 1.8 | 1.9 | 1.7 | 1.2 | 3.1 | 6.8 | 3.9 | 2 |
| 36 | 1.6 | 1.1 | 1.2 | 1 | 1.2 | 0.9 | 8.4 | 4.9 | 2 |
| 37 | 3.1 | 2 | 3.2 | 1.7 | 2.2 | 7.3 | 3.6 | 91.4 | 2 |
| 38 | 2.6 | 2.2 | 2.9 | 1.7 | 1.8 | 4.7 | 10.5 | 146 | 2 |
| 39 | 3.3 | 1 | 2.7 | 1.5 | 2 | 1.7 | 10.1 | 3.9 | 2 |
| 40 | 2.8 | 1.2 | 2.6 | 1.7 | 2.2 | 5.5 | 13.4 | 16.9 | 1 |
| 41 | 2.4 | 1.4 | 1.3 | 1.4 | 1.7 | 3.2 | 1.1 | 6.3 | 1 |
| 42 | 1.6 | 1.7 | 1.2 | 1.5 | 1.1 | 4.6 | 19.7 | 7.5 | 1 |
| 43 | 1 | 1.2 | 1.7 | 1.1 | 1.1 | 3.6 | 9 | 2.6 | 1 |
| 44 | 2.2 | 1.4 | 1.6 | 1.6 | 1.5 | 5.7 | 0.7 | 7.8 | 2 |
| 45 | 2.6 | 2.5 | 2.2 | 1.5 | 1.5 | 7.5 | 19.1 | 18.3 | 2 |
| 46 | 1.9 | 1 | 1.8 | 1.4 | 1.1 | 1.9 | 16.1 | 17 | 2 |
| 47 | 2 | 1.8 | 1.5 | 1.3 | 1.2 | 10.8 | 8 | 6.1 | 2 |
| 48 | 1.7 | 1.5 | 1.4 | 1.3 | 1.9 | 7.4 | 9.9 | 21.5 | 2 |

* Individual average value of scores in 10 fields

** Average total value in 10 fields

*** (1) negative in cases <1% and (2) positive in cases >1%.

**Supplementary Table 4. Impactor of histological grade in TIME**

| **Antibody** | **Histological grading** | **N** | **Mean*** | **Standard deviation** | **p-value** |
| --- | --- | --- | --- | --- | --- |
| CD3 | Well differentiated | 16 | 2.375 | 0.6424 | 0.763 |
|  | Moderately differentiated | 25 | 2.264 | 0.5715 |  |
|  | Poorly differentiated | 7 | 2.271 | 0.5619 |  |
| CD4 | Well differentiated | 16 | 1.506 | 0.4171 | 0.411 |
|  | Moderately differentiated | 25 | 1.712 | 0.5395 |  |
|  | Poorly differentiated | 7 | 1.714 | 0.4811 |  |
| CD8 | Well differentiated | 16 | 2.069 | 0.6118 | 0.596 |
|  | Moderately differentiated | 25 | 1.924 | 0.5426 |  |
|  | Poorly differentiated | 7 | 1.829 | 0.5499 |  |
| CD20 | Well differentiated | 16 | 1.688 | 0.4745 | 0.485 |
|  | Moderately differentiated | 25 | 1.696 | 0.5820 |  |
|  | Poorly differentiated | 7 | 1.486 | 0.5521 |  |
| CD68 | Well differentiated | 16 | 2.006 | 0.6169 | 0.237 |
|  | Moderately differentiated | 25 | 1.756 | 0.4700 |  |
|  | Poorly differentiated | 7 | 1.600 | 0.4830 |  |
| CD83 | Well differentiated | 16 | 6.738 | 3.4842 | 0.23 |
|  | Moderately differentiated | 25 | 5.636 | 4.1819 |  |
|  | Poorly differentiated | 7 | 4.829 | 3.2520 |  |
| CD1a | Well differentiated | 16 | 9.544 | 3.9623 | 0.925 |
|  | Moderately differentiated | 25 | 9.512 | 6.5455 |  |
|  | Poorly differentiated | 7 | 9.471 | 2.9753 |  |
| PD-1 | Well differentiated | 16 | 28.256 | 37.6193 | 0.456 |
|  | Moderately differentiated | 25 | 15.000 | 12.7770 |  |
|  | Poorly differentiated | 7 | 16.071 | 12.5833 |  |
| PD-L1 | Well differentiated | 16 | 1.994 | 0.9270 | 0.747 |
|  | Moderately differentiated | 25 | 1.848 | 0.9283 |  |
|  | Poorly differentiated | 7 | 1.829 | 0.5880 |  |
|  |  |  |  |  |  |
| *Teste de Kruskal-Wallis |  |  |  |  |  |
